# Supplementary material for: LSTM-based sentiment analysis for stock price forecast
Source: PeerJ Comput Sci. 2021 Mar 11;7:e408. doi: 10.7717/peerj-cs.408 (PMC7959635; doi:10.7717/peerj-cs.408)
Supplement: Supplemental Information 1 — sentiment/ The directory including training variation testing data of sentiment analysis in Chinese Using BERT. Codes are also included. BERT Pre-train model is not included. sentiment/data/ The directory including the datasets sentiment/train.sh: Training sentiment/predict.sh: Testing sentiment/intent.py: Using model for single sentence inference sentiment/chinese_-12_H-768_A-12: pretrain model from google (not included) stockPrice/ The directory including training variation testing data of stock information from TWSE open data. Codes are also included. stockPrice/data/ The directory including the datasets of stock information stockPrice/prediction.py Codes of this article. [file peerj-cs-07-408-s001.zip › Codes/Read.me.rtf]

sentiment/  Including training variation testing data of sentiment analysis in Chinese Using BERT. Codes are also included. BERT Pre-train model is not included stockPrice/ Including  training variation testing data of stock information from TWSE open data. Codes are also included.
